# Parallel loss of introns in the *ABCB1* gene in angiosperms

**DOI:** 10.1186/s12862-017-1077-x

**Published:** 2017-12-04

**Authors:** Rajiv K. Parvathaneni, Victoria L. DeLeo, John J. Spiekerman, Debkanta Chakraborty, Katrien M. Devos

**Affiliations:** 1 Institute of Plant Breeding, Genetics and Genomics, University of Georgia, 30602 Athens, Georgia United States; 2 Department of Genetics, University of Georgia, 30602 Athens, GA United States; 3 Department of Plant Biology, University of Georgia, 30602 Athens, GA United States; 4 Institute of Bioinformatics, University of Georgia, 30602 Athens, GA United States; 5 Current address: Donald Danforth Plant Science Center, St. Louis, MO 63132 United States; 6 Current address: Department of Biology, Pennsylvania State University, University Park, PA 16802 United States

**Keywords:** Intron loss, Parallel intron loss, *ABCB1* gene, Gene structure, Grasses, Monocots, Angiosperms, Conserved motif

## Abstract

**Background:**

The presence of non-coding introns is a characteristic feature of most eukaryotic genes. While the size of the introns, number of introns per gene and the number of intron-containing genes can vary greatly between sequenced eukaryotic genomes, the structure of a gene with reference to intron presence and positions is typically conserved in closely related species. Unexpectedly, the *ABCB1* (ATP-Binding Cassette Subfamily B Member 1) gene which encodes a P-glycoprotein and underlies dwarfing traits in maize (*br2*), sorghum (*dw3*) and pearl millet (*d2*) displayed considerable variation in intron composition.

**Results:**

An analysis of the *ABCB1* gene structure in 80 angiosperms revealed that the number of introns ranged from one to nine. All introns in *ABCB1* underwent either a one-time loss (single loss in one lineage/species) or multiple independent losses (parallel loss in two or more lineages/species) with the majority of losses occurring within the grass family. In contrast, the structure of the closest homolog to *ABCB1*, *ABCB19*, remained constant in the majority of angiosperms analyzed. Using known phylogenetic relationships within the grasses, we determined the ancestral branch-points where the losses occurred. Intron 7, the longest intron, was lost in only a single species, *Mimulus guttatus*, following duplication of *ABCB1*. Semiquantitative PCR showed that the *M. guttatus ABCB1* gene copy without intron 7 had significantly lower transcript levels than the gene copy with intron 7. We further demonstrated that intron 7 carried two motifs that were highly conserved across the monocot-dicot divide.

**Conclusions:**

The *ABCB1* gene structure is highly dynamic, while the structure of *ABCB19* remained largely conserved through evolution. Precise removal of introns, preferential removal of smaller introns and presence of at least 2 bp of microhomology flanking most introns indicated that intron loss may have predominantly occurred through non-homologous end-joining (NHEJ) repair of double strand breaks. Lack of microhomology in the exon upstream of lost phase I introns was likely due to release of the selective constraint on the penultimate base (3rd base in codon) of the terminal codon by the splicing machinery. In addition to size, the presence of regulatory motifs will make introns recalcitrant to loss.

**Electronic supplementary material:**

The online version of this article (10.1186/s12862-017-1077-x) contains supplementary material, which is available to authorized users.

## Background

Introns are a characteristic and common feature in eukaryotic genomes. They likely accumulated very early in eukaryotic evolution and some introns have remained in conserved positions across kingdoms for a period close to two billion years [1–3]. Although it is conceivable that positionally conserved introns arose independently through insertions in the same location in orthologous genes, simulation studies and estimations of the probability of parallel gains for individual introns have indicated that the majority of shared introns likely have a common origin [1, 2, 4].

Evolutionary loss and gain of introns in genomic sequence data may provide a mechanism by which organisms diversify gene expression and gene function. The rate of gain and loss of introns varies with the lineage but in most cases intron loss is higher, sometimes by a few orders of magnitude, than intron gain [5–9]. Rates of intron loss have been calibrated in humans at 4–5 × 10^−10^ per intron per year by Roy and Gilbert [5] and 4.28 × 10^−13^ by Coulombe-Huntington and Majewski [6]. Similar rates of intron loss have been observed in plants, including *Arabidopsis thaliana* (1–3 × 10^−10^ [8]), *A. lyrata* (2.73 × 10^−11^ [8]), *Oryza sativa* (3.3 × 10^−10^ [10]; 8.1 × 10^−11^ [9]), and the grasses *Setaria italica, Brachypodium distachyon, Sorghum bicolor* and *Zea mays* (1.1–1.8 × 10^−10^ [9]).

Two main mechanisms for intron loss have been proposed, that is reverse transcription (RT)-mediated intron loss and intron deletion triggered by repair of double strand breaks (DSBs) via non-homologous end joining (NHEJ). RT-mediated loss occurs when a cDNA recombines with its genomic copy. Characteristics of RT-mediated loss include precise removal of introns, preferential removal of small introns, deletion of adjacent introns and a 3′ bias of intron removal [11–13]. A signature of genomic deletion of introns via NHEJ is the presence of 2–8 bp of micro-homology. Intron removal through NHEJ can be precise or leave a footprint. If DSB repair occurs through microhomology between splice sites, intron removal via NHEJ will be precise [14]. Because the chance that microhomology is encountered elsewhere in an intron increases with increasing intron length, precise removal of introns through NHEJ is expected to occur at higher frequencies for short introns [14]. The presence of short direct repeats flanking deleted introns has been observed in *A. thaliana* and *A. lyrata*, *Caenorhabditis elegans* and *Drosophila* [8, 15, 16]. The latter two species also show relatively high frequencies of imprecise intron removal supporting the role of NHEJ in intron removal. Overall, the jury is still out as to whether RT- or NHEJ- meditated removal is the dominant mechanism of intron loss. Furthermore, which DSB repair pathways is used may vary by species [17].

Intron loss has been documented in whole genome studies of multiple species. Comparison of 10 fully sequenced genomes within the genus *Drosophila* uncovered a total of 1754 intron loss events [6]. A comparison between the genome sequences of *A. thaliana* and *A. lyrata* revealed a combined loss of 105 introns [8], and a similar study across five sequenced grass genomes (maize, sorghum, rice, foxtail millet and *Brachypodium*) using *Arabidopsis* and banana as outgroups revealed a total of 745 intron loss events, including 93 cases of parallel intron loss whereby the same intron was lost independently in multiple lineages [9]. Until the latter study, only a few cases of parallel intron loss had been described in the literature. The glyceraldehyde-3-phosphate dehydrogenase (*GAPDH*) gene which has 10 introns showed parallel loss of intron 9 in opossum, dog and the primate/rodent lineages [11]. The *white* gene in the distantly related dipteran (true fly) lineages contains 14 introns, three of which (introns 10, 11 and 13) underwent parallel loss 3, 4 and 5 times, respectively [18]. In the *Drosophila* and mosquito species, convergent loss of one intron, intron Z, was observed in the *MRP1* gene [19]. Incidentally, two of the three genes for which parallel intron loss has been reported in animals, *white* and *MRP1,* are ABC transporters. In Angiosperms, the isochorismate synthase gene, which comprises 15 introns, underwent parallel loss of intron 2 in the grass lineage and the *Medicago truncatula/Glycine max* lineage [20]. The overall characteristics of introns that have been lost once and those that have been lost multiple times are very similar [9].

In the current study, we conducted a detailed analysis of intron loss in the *ABCB1* gene across Angiosperms. *ABCB1* encodes a P-glycoprotein that modulates basipetal auxin transport in stems [21]. The protein comprises two transmembrane domains (TMDs) and two nucleotide binding domains (NBDs) [22]. Mutations in *ABCB1* in grasses significantly reduce plant height and these mutants have been exploited extensively in agriculture to reduce lodging under high inputs and hence to increase yields [23–25]. Examples of *ABCB1* mutants of global economic importance are sorghum *dw3* and pearl millet *d2* [23, 24]. Intron loss in *ABCB1* was first observed when pearl millet *ABCB1* was isolated as a candidate for the *d2* dwarfing trait [23] and compared with sorghum *ABCB1*. We subsequently expanded the structural analysis of the *ABCB1* gene to other sequenced and non-sequenced members of the grasses and beyond, including selected non-grass monocots, dicots and the basal species *Amborella trichopoda*.

## Methods

### Retrieval of protein, cDNA and gene sequences

A BLASTP analysis was performed using *Arabidopsis* ABCB1 (locus AT2G36910) and ABCB19 (locus AT3G28860) proteins as queries against plant proteins in Phytozome 11.0 (phytozome.jgi.doe.gov/), Gramene (www.gramene.org) and the non-redundant protein section of GenBank (www.ncbi.nlm.nih.gov). In first instance, proteins with >70% similarity to *Arabidopsis* ABCB1 and >75% similarity to *Arabidopsis* ABCB19 were considered ‘true’ orthologs of ABCB1 and ABCB19, respectively, and were retrieved for analysis together with their corresponding genomic and cDNA sequences (Additional file 1: Table S1). Orthology of the retrieved sequences was confirmed by reciprocal blast analyses at the nucleotide level using *Arabidopsis ABCB1* and rice *ABCB1* for dicot and monocot *ABCB1* sequences, respectively. A protein in banana (*Musa acuminata*) had 67% similarity to *Arabidopsis* ABCB19 and was also included in the analysis. Because no ABCB1 protein homolog was identified in *Carica papaya*, a BLASTN analysis using *Arabidopsis ABCB1* cDNA as query was performed against the genome sequence of *C. papaya*. We found that the *ABCB1* gene in *C. papaya* (evm.TU.supercontig_102.36) had been misannotated due to gaps in the sequence but was likely full-length. This *ABCB1* homolog was included in the gene structure analysis. *ABCB1* gene sequences from additional monocot species for which whole or partial genome sequence was obtained prepublication (sources indicated in Additional file 1: Table S1) were identified by reciprocal BLASTN analyses using the rice (LOC_Os08g45030) or sorghum (AY372819) *ABCB1* cDNA sequence as query. Top hits in reciprocal blast searches were considered true orthologs. A total of 81 *ABCB1* sequences from 27 monocot species, 32 dicot species and 1 basal angiosperm, and 84 *ABCB19* sequences from 20 monocot and 32 dicot species were analyzed. The selected ABCB1 and ABCB19 protein sequences were aligned using MUSCLE v3.8.31 with default settings [26]. *Arabidopsis* ABCB4 (AT2G47000) was included as outgroup. We used either the proteins retrieved from the various databases or, when the protein structure appeared to be incorrect, *in silico* translations from cDNAs generated from genomic sequence alignments in the protein alignment. *Phyllostachys edulis* and *Chasmanthium laxum* were not translated *in silico* because of gaps in the *ABCB1* coding sequence. A maximum likelihood tree was constructed from the MUSCLE alignment using RAxML v.8.2.4 [27] (parameters -m PROTGAMMAWAGF, −× 12,345, −# 100) to further confirm orthology of the sequences used in our analyses. FigTree v1.2.2. was used to visualize the phylogenetic tree [28].

### Sequence alignment and determination of intron sites

The retrieved *ABCB1* and *ABCB19* genomic sequences were aligned at the nucleotide level using MUSCLE v3.8.31 with default settings [26]. Because of the large number of entries, sequence alignments were done separately for monocot *ABCB1*, dicot *ABCB1*, monocot *ABCB19* and dicot *ABCB19*. Where necessary, sequence alignments were edited manually using Jalview [29]. A sequence alignment between genomic DNA and cDNA of *ABCB1* and *ABCB19* of *Arabidopsis* and rice identified the introns in each of the genes and their correspondence across the two genes, allowing conclusions to be drawn from the separate alignments on the differential presence of introns across species and genes.

### Plant material and DNA extractions

Seeds of the grass species *Danthoniopsis dinteri*, *Sacciolepis myosuroides* and *Arundinella hirta*, and leaf material from *Brachiaria spp.*, *Andropogon gerardii, Bambusa* spp. and *Acroceras macrum* were obtained from Melanie L. Harrison, Plant Genetic Resources Conservation Unit (PGRCU), Griffin, GA. The seeds were planted in 3 in. pots and grown in a greenhouse under natural light and day/night temperatures of 32 °C/28 °C. *Paspalum vaginatum* cultivar ‘SeaDwarf’ was provided as a clonal propagule by Paul Raymer, University of Georgia (UGA). Leaves from the Zoysia grass cultivar ‘Emerald’ (*Zoysia japonica* X *Zoysia pacifica*) were collected from the lawn outside the UGA Miller Plant Sciences building. Leaves from a *Phyllostachys* spp. were collected from a bamboo stand on the UGA North Campus. Leaves from *Typha latifolia* were collected from the UGA Plant Biology greenhouse teaching collection. Leaves from *Pharus mezhii* and *Streptochaeta* spp., and DNA from *Anomochloa* spp., *Phragmites australis* and *Micraira subulifolia* were provided by Elizabeth Kellogg, Donald Danforth Plant Science Center, St. Louis, Missouri. DNA was extracted from leaf samples using the Qiagen DNA extraction kit. DNA of *Mayaca* spp. and *Ecdieocolea* spp. was provided by Jim Leebens-Mack (UGA), of *Zea mays* by Kelly Dawe (UGA) and of *Oryza sativa* spp. *japonica* (Nipponbare) by Jeff Bennetzen (UGA).

### Primer design and PCR amplification across introns

Using the MUSCLE alignment of the monocot *ABCB1* gene sequences, primer sets were designed against conserved exon regions to amplify the introns in a range of monocot species. The primer pairs were developed to amplify across introns 1 + 2, 3 + 4, 5, 6, 7, 8 and 9 (Table 1). PCR amplifications were performed in 20 μl reaction volumes containing 1X PCR buffer (GoTaq buffer), 1.5 mM MgCl_2_, 0.25 mM of each dNTP, 0.5 μM forward and reverse primers, 10–25 ng of DNA template and 1 U of Taq DNA Polymerase (Promega, Madison, WI). Amplification conditions consisted of initial denaturation at 95 °C for 5 min followed by 34 cycles of 95 °C for 30 s, the appropriate Tm (see Table 1) for 30 s and 72 °C for 2 min, and a final extension of 72 °C for 10 min. PCR products were separated on 0.8% agarose gels. Amplicon sizes were used as a proxy for the presence and absence of introns. DNA from the sequenced genomes rice, sorghum and maize was used as positive control. A selection of amplicons was Sanger sequenced to validate the PCR results.

**Table 1:** List of primers used to amplify across different introns in *ABCB1*

Primers	Sequence	Annealing temp. (°C)	Primer location
Forward			
ABCB1_F5	GACCTCTTCCGCTTCGCCGACG	61	Exon 1
ABCB1_F6	TTCCTCCGCTTCTTCGCCGACCTCG	61	Exon 1
ABCB1_F8	CGCCTCGTCGTCAAGTACGCCTTC	61	Exon 1- Exon 2 boundary
ABCB1_F22	GACGTCATCTACGCCATCAACGC	61	Exon 3
ABCB1_F16	ACCTACTTCACCGTCTTCTGCTG	55	Exon 5
ABCB1_F17–1	TCCGGGTCAGGGAAGAGCAC	61	Exon 6
ABCB1_FP2	GCTCATCGAGAGGTTCTACGA	57	Exon 6
ABCB1_F27	CAGGAGCCGACGCTGTTCGC	61	Exon 7
ABCB1_F26	CATCAAGGAGAACCTGCTGCTGGG	61	Exon 7
ABCB1_FP1	CATCAAGGAGAACCTGCTGCT	57	Exon 7
ABCB1_F25	GGCGCTGGACCGCTTCATGAT	61	Exon 8
ABCB1_F24	GCCACCAGCGCGCTGGAC	61	Exon 8
ABCB1_F29	CTTCAGCGCCATCTTCGCCTAC	61	Exon 9
Reverse			
ABCB1_R9	GCTTCTCGCTGATGGCGTCCTG	61	Exon 3
ABCB1_R20	TAGCAGCAGAAGACGGTGAAGTAGGT	55	Exon 5
ABCB1_R16–2	GCCATGCTCGGAGCCGACT	61	Exon 6
ABCB1_R19–1	TCCCCAGCAGCAGGTTCTC	61	Exon 7
ABCB1_R25	GCGCTGGTGGCCTCGTCCA	61	Exon 8
ABCB1_R26	CGGTCCAGCGCCTCCTGCA	61	Exon 8
ABCB1_RP1A	GATGGCGATGCGCTGCTTCTG	57	Exon 8
ABCB1_RP1B	GATGGCGATCCGCTGCTTCTG	57	Exon 8
ABCB1_RP2	GAAGATGGCGCTGAAGGAGCC	57	Exon 9
ABCB1_R29	CCGATGAGGAGTAGCAGTA	61	Exon 9
ABCB1_R27	ACGTAGGCGAAGATGGCGCT	61	Exon 9
ABCB1_R1	GCGTASGASGCGTASAGSAGGAACTG	61	Exon 10

### Long-range PCR to isolate and validate the gene structure

Full-length *ABCB1* gene isolation and sequencing were performed in the species *Ecdeiocolea* spp., *Mayaca* spp., *Typha latifolia*, *Streptochaeta* spp., *Phyllostachys* spp., *Paspalum vaginatum* and *Zoysia* spp. to verify that fragment lengths of PCR amplicons had been correctly interpreted. The six species were selected because of their phylogenetic position and/or their unusual intron composition. Several sets of primers were designed against conserved regions, identified based on the multiple sequence alignment (MSA), in the first and last exons of *ABCB1* (Table 2). The primer combination ABCB1F1/R1 amplified *ABCB1* from the majority of the selected species. *Zoysia* and *Mayaca* required the use of different forward/reverse primers (Table 2). Long-range PCR was performed in 12.5 μl reaction volumes comprising 0.4 μM forward primer, 0.4 μM reverse primer, 0.3 mM of each dNTP, 1X Long Amp buffer and 1.25 U of Long Amp Taq DNA polymerase (New England Biolabs, Ipswich, MA). The PCR conditions were as follows: Pre-heat the PCR block to 95 °C; 95 °C for 30 s for initial denaturation followed by 35 cycles of 95 °C for 30 s, 61 °C for 30 s and 68 °C for 6 min, and a final extension at 68 °C for 20 min. Amplification products were checked on a 0.8% agarose gel. Products with sizes greater than 3 kb were either cleaned with a QIAquick PCR purification kit (Qiagen, Valencia, CA) or gel extracted using a GeneJet gel extraction kit (Life Technologies, Grand Island, NY). PCR products were cloned in the pGEM-T vector (Promega, Madison, WI) and Sanger sequenced.

**Table 2:** List of primers used for long-range PCR across all *ABCB1* introns

Primers	Sequence	Annealing temp. (°C)	Species in which primers were successfully used
Forward			
ABCB1_F5	GACCTCTTCCGCTTCGCCGACG	61	*Zoysia*
ABCB1_F1	CGCTTCTTYGCSGABCTBGTSGACTC	61	*Phyllostachys, Typha, Ecdieocolea, Mayaca, Paspalum, Streptochaeta*
Reverse			
ABCB1_R4	CCGTGATCTTGCGCTCCGCGTTG	61	*Zoysia*
ABCB1_R1	GCGTASGASGCGTASAGSAGGAACTG	61	*Phyllostachys, Typha, Ecdieocolea, Paspalum, Streptochaeta*
ABCB1_R22	GACACCATCAGCACCATGAA	61	*Mayaca*

### Motif finding

The intron 7 sequences of 81 *ABCB1* orthologs from 27 monocots, 32 dicots and the basal angiosperm *Amborella trichopoda* were used to identify motifs using the ‘Multiple EM for Motif Elicitation’ (MEME) version 4.10.1 (http://meme-suite.org/tools/meme) [30]. Default settings were used except that the number of motifs was set to 10, minimum and maximum widths were set to 12, and the distribution of motif sites was set to One Occurrence Per Sequence (oops). To test the probability of finding a motif using these parameters in sequences of similar length to intron 7, we randomly selected a gene in each of 10 species (five grasses: *Brachypodium distachyon, Oryza sativa, Sorghum bicolor, Zea mays, Setaria italica;* one non-grass monocot: *Musa acuminata*; four dicots: *A. thaliana*, *Glycine max, Populus trichocarpa and Phaseolus vulgaris*) and extracted 1080 bp upstream of the start codon. A MEME analysis was conducted on this set of 10 sequences. This was repeated 100 times using different genes for each analysis. A similar analysis was conducted with 1080 bp of randomly selected genic sequence. In addition, we conducted MEME analyses on intron 9 (median length: 1266 bp) and on concatenated introns 1, 2, 4, 5, 6 and 7 (median length: 1073 bp) across 59 *ABCB19* genes as well as on *ABCB1* intron 7 across the same 59 species (median intron length: 1084 bp). The first five and last five bp of each intron were removed before concatenation to remove the conserved splice sites.

### Semiquantitative PCR

cDNA from leaves, buds and flowers (three biological replicates) and genomic DNA from *Mimulus guttatus* inbred lines IM62 and IM767 were provided by Andrea Sweigart (UGA). Primers were designed targeting the two *ABCB1* homologs Migut.J00652 (copy containing intron 7; primers MimL01707-F1: 5′-GGCTTGCACTTGTTCTGA-3′, and MimL01707-R2: 5′-CGGTTGCAGACTGGCGAT-3′) and Migut.L01707 (copy without intron 7; primers MimJ00652-F1: 5′-GCCTCGCTCTAGTTCTTG-3′, and MimJ00652-R4: 5′-AGTTTGCAGGCTCGACGT-3′) to amplify a 212 bp fragment from cDNA, and 308 bp (Migut.J00652) and 298 bp (Migut.L0107) fragments from genomic DNA. Amplification with actin primers MgACTIN.F (5′-ATGGTAACATTGTGCTCAGTGGT-3′) and MgACTIN.R (5′-GATAGAACCTCCAATCCAGACACTGTA-3′) [31] was used as control. Semiquantitative PCR was conducted in 25 μl reaction volumes containing 1X GoTaq^®^ Flexi PCR buffer (Promega), 1.5 mM MgCl_2_, 0.25 mM of each dNTP, 0.5 μM forward and reverse primers, 1 U GoTaq DNA polymerase (Promega) and 1 μl of a 1:10 cDNA dilution. PCR conditions were as follows: initial denaturation at 95 °C for 2 min followed by 31 cycles (Migut.J00652 and Migut.L01707) or 26 cycles (Actin) of denaturation at 95 °C for 30 s, annealing at 50 °C for 30 s and extension at 72 °C for 1 min, followed by a final extension at 72 °C for 5 min. PCR products were separated on a 1.5% agarose gel. The same reaction and PCR conditions were used for amplification from genomic DNA using 20 ng of DNA as input.

## Results

### Structural analysis of *ABCB1* and *ABCB19* across sequenced genomes

**Fig. 1:**
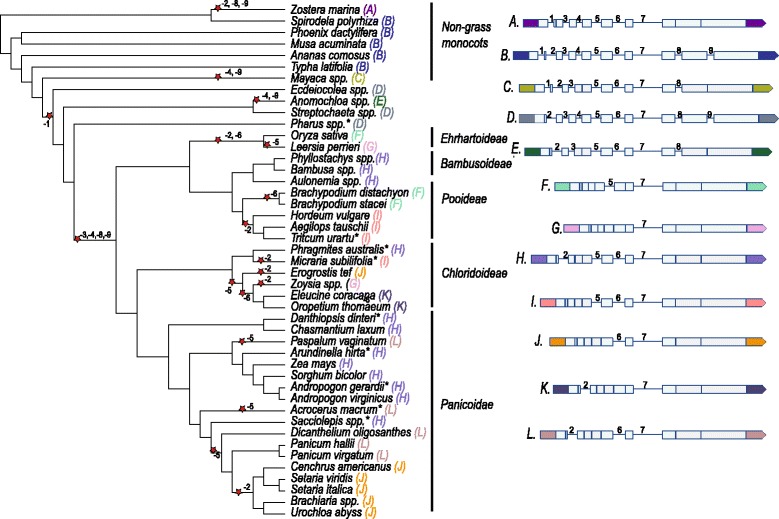
Schematic representation of the gene structure variation in *ABCB1* in selected members of the grass family and non-grass monocots. The 5′ and 3′ UTR regions and species names are color-coded to match. Species with the same color code and the same letter in parenthesis behind the species name have the same intron composition. Intron loss is shown by a red star and a minus (−) sign. The figure is not drawn to scale

All analyzed *ABCB1* and *ABCB19* genes fell into two distinct clades (Additional file 2). *ABCB1* is present in one copy per monoploid genome in most plant species. Diploids thus carry a single *ABCB1* gene while polyploids [32–46] likely carry multiple copies. The copy number in polyploids depends on the number of rounds of whole genome duplication and the extent of subsequent diploidization that has occurred in the species. An initial comparison of pearl millet *ABCB1* (*Ca_ABCB1*) with *ABCB1* in the panicoid species sorghum (*Sb_ABCB1*; AY372819.1), maize (*Zm_ABCB1*; GRMZM2G315375) and foxtail millet (*Si_ABCB1*; Seita.6G253500) had revealed that *ABCB1* in pearl millet and foxtail millet carried two introns while *ABCB1* in maize and sorghum carried four introns (Fig. 1; Table 3). Analysis of *ABCB1* in *Brachypodium* and rice revealed further variation in the *ABCB1* gene structure (Fig. 1; Table 3). Interestingly, while the number of introns in grasses appeared to be limited to four, the *Arabidopsis ABCB1* gene carried nine introns. Expanding the *ABCB1* comparison to all sequenced genomes available at the time demonstrated that 28 out of the 32 dicots analyzed, four of the five non-grass monocots analyzed, but none of the *Poaceae* species had nine introns (Table 3; Additional files 3 and 4). *ABCB1* in the dicots *Eutrema salsugineum* (Thhalv10016150m.g) and *Cucumis sativus* (Cusca.306720), and one of the two *ABCB1* copies in *Mimulus guttatus* (Migut.L01707) had eight introns and lacked introns 8, 9 and 7, respectively (Table 3). *Linum usitatissimum* carried two *ABCB1* copies, but one gene copy (Lus10014427.g) carried two genomic deletions, one that spanned the region from the 3′ end of exon 2 to the 5′ end of exon 4, and one that spanned part of exon 7 and part of intron 7. Lus10014427.g is likely a pseudogene and was removed from the analysis. The presumed functional *ABCB1* copy in *L. usitatissimum* (Lus10023929.g) lacked intron 2. *Zostera marina*, a seagrass which belongs to the order *Alismatales*, lacked introns 2, 8 and 9. The number of introns in *ABCB1* in grasses varied from 1 to 4. All sequenced grass species carried intron 7, lacked introns 1, 3, 4, 8 and 9, and had varying combinations of introns 2, 5 and 6 (Table 3). In all cases, intron loss was precise.

**Table 3:** Presence/absence polymorphisms of introns in *ABCB1* in select angiosperms

		Intron number									Methodology^a^
Species	Locus ID	1	2	3	4	5	6	7	8	9	
Dicots		Intron presence/absence^b^									
*Fabaceae*											
*Phaseolus vulgaris*	Phvul.001G179300	P^b^	P	P	P	P	P	P	P	P	Seq
*Phaseolus vulgaris*	Phvul.007G147400	P	P	P	P	P	P	P	P	P	Seq
*Medicago truncatula*	Medtr7g102070	P	P	P	P	P	P	P	P	P	Seq
*Medicago truncatula*	Medtr1g063170	P	P	P	P	P	P	P	P	P	Seq
*Trifolium pratense*	Tp57577_TGAC_v2_gene32830	P	P	P	P	P	P	P	P	P	Seq
*Glycine max*	Glyma.19G184300	P	P	P	P	P	P	P	P	P	Seq
*Glycine max*	Glyma.03G183600	P	P	P	P	P	P	P	P	P	Seq
*Glycine max*	Glyma.10G055000	P	P	P	P	P	P	P	P	P	Seq
*Glycine max*	Glyma.13G142100	P	P	P	P	P	P	P	P	P	Seq
*Rosaceae*											
*Prunus persica*	Prupe.7G129600	P	P	P	P	P	P	P	P	P	Seq
*Malus domestica*	MDP0000231597	P	P	P	P	P	P	P	P	P	Seq
*Malus domestica*	MDP0000183294	P	P	P	P	P	P	P	P	P	Seq
*Fragaria vesca*	gene09601-v1.0-hybrid	P	P	P	P	P	P	P	P	P	Seq
*Cucurbitaceae*											
*Cucumis sativus*	Cucsa.306720	P	P	P	P	P	P	P	P	A	Seq
*Rutaceae*											
*Citrus clementina*	Ciclev10010916m.g	P	P	P	P	P	P	P	P	P	Seq
*Citrus sinensis*	orange1.1g000687m.g	P	P	P	P	P	P	P	P	P	Seq
*Myrtaceae*											
*Eucalyptus grandis*	Eucgr.K02930	P	P	P	P	P	P	P	P	P	Seq
*Malvaceae*											
*Theobroma cacao*	Thecc1EG022244	P	P	P	P	P	P	P	P	P	Seq
*Gossypium raimondii*	Gorai.002G246800	P	P	P	P	P	P	P	P	P	Seq
*Gossypium raimondii*	Gorai.006G163000	P	P	P	P	P	P	P	P	P	Seq
*Brassicaceae*											
*Eutrema salsugineum*	Thhalv10016150m.g	P	P	P	P	P	P	P	A	P	Seq
*Capsella grandiflora*	Cagra.0558 s0007	P	P	P	P	P	P	P	P	P	Seq
*Capsella rubella*	Carubv10022511m.g	P	P	P	P	P	P	P	P	P	Seq
*Boechera stricta*	Bostr.23794 s0400	P	P	P	P	P	P	P	P	P	Seq
*Brassica rape FPsc*	Bra023087	P	P	P	P	P	P	P	P	P	Seq
*Brassica rapa FPsc*	Bra017216	P	P	P	P	P	P	P	P	P	Seq
*Brassica oleracea*	Bo3g031410	P	P	P	P	P	P	P	P	P	Seq
*Brassica oleracea*	Bo4g186480	P	P	P	P	P	P	P	P	P	Seq
*Arabidopsis lyrata*	934,330	P	P	P	P	P	P	P	P	P	Seq
*Arabidopsis thaliana columbia*	AT2G36910	P	P	P	P	P	P	P	P	P	Seq
*Caricaceae*											
*Carica papaya*	evm.TU.supercontig_102.36	P	P	P	P	P	P	P	ND	P	Seq
*Amaranthaceae*											
*Chenopodium quinoa*	AUR62040338	P	P	P	P	P	P	P	P	P	Seq
*Chenopodium quinoa*	AUR62042509	P	P	P	P	P	P	P	P	P	Seq
*Euphorbiaceae*											
*Ricinus communis*	30,078.t000079	P	P	P	P	P	P	P	P	P	Seq
*Manihot esculenta*	Manes.09G065700	P	P	P	P	P	P	P	P	P	Seq
*Manihot esculenta*	Manes.08G011100	P	P	P	P	P	P	P	P	P	Seq
*Linaceae*											
*Linum usitatissimum*	Lus10023929.g	P	A	P	P	P	P	P	P	P	Seq
*Salicaceae*											
*Salix purpurea*	SapurV1A.1230 s0070	P	P	P	P	P	P	P	P	P	Seq
*Salix purpurea*	SapurV1A.0584 s0030	P	P	P	P	P	P	P	P	P	Seq
*Populus trichocarpa*	Potri.006G123900	P	P	P	P	P	P	P	P	P	Seq
*Populus trichocarpa*	Potri.016G093600	P	P	P	P	P	P	P	P	P	Seq
*Solanaceae*											
*Solanum lycopersicum*	Solyc09g008240.2	P	P	P	P	P	P	P	P	P	Seq
*Solanum tuberosum*	PGSC0003DMG400003889	P	P	P	P	P	P	P	P	P	Seq
*Phrymaceae*											
*Mimulus guttatus*	Migut.J00652	P	P	P	P	P	P	P	P	P	Seq
*Mimulus guttatus*	Migut.L01707	P	P	P	P	P	P	A	P	P	Seq
*Ranunculaceae*											
*Aquilegia coerulea Goldsmith*	Aquca_030_00072	P	P	P	P	P	P	P	P	P	Seq
*Aquilegia coerulea Goldsmith*	Aquca_062_00022	P	P	P	P	P	P	P	P	P	Seq
Non-grass monocots (except Poales)											
*Zostera marina*	Zosma16g00300	P	A	P	P	P	P	P	A	A	Seq
*Spirodela polyrhiza*	Spipo1G0026900	P	P	P	P	P	P	P	P	P	Seq
*Phoenix dactylifera*	LOC103714888	P	P	P	P	P	P	P	P	P	Seq
*Phoenix dactylifera*	LOC103706998	P	P	P	P	P	P	P	P	P	Seq
*Musa acuminata*	GSMUA_Achr4G12130_001	P	P	P	P	P	P	P	P	P	Seq
*Musa acuminata*	GSMUA_Achr4G04430_001	P	P	P	P	P	P	P	P	P	Seq
Poales (except Poaceae)											
*Ananas comosus*	Aco010196	P	P	P	P	P	P	P	P	P	Seq
*Typha latifolia*	Not assigned	P	P	P	P	P	P	P*	P	P	LR-PCR/Seq
*Ecdeiocolea* spp.	Not assigned	A	P	P	P	P	P	P	P	P	LR-PCR/Seq
*Mayaca* spp.	Not assigned	P	P	P	A	P	P	P	P	A	LR-PCR/Seq
Poaceae											
*Streptochaeta* spp.	Not assigned	A	P	P	P	P	P	P	P	P	LR-PCR/Seq
*Anomochloa* spp.	Not assigned	A*	P*	P*	A*	ND	P	P*	P	A	PCR/Seq
*Pharus mezhii*	Not assigned	A*	P*	P	P*	P	P*	ND	P	ND	PCR/Seq
*Leersia perrieri*	LPERR08G20590	A	A	A	A	A	A	P	A	A	Seq
*Oryza sativa* ssp. *indica*	BGIOSGA029208	A	A	A	A	P	A	P	A	A	Seq
*Oryza sativa* ssp. *japonica*	LOC_Os08g45030	A	A	A	A	P	A	P	A	A	Seq
*Aulonemia* spp.	Not assigned	A*	P*	A*	A*	P	ND	P	A	ND	PCR/Seq
*Bambusa* spp.	Not assigned	A	P	A	A	P	P	P*	A	A	PCR/Seq
*Phyllostachys* spp.	Not assigned	A	P	A	A	P	P	P	A	A	LR-PCR/Seq
*Phyllostachys edulis*	PH01000979	A	P	A	A	P	P	P	A	A	Seq
*Phyllostachys edulis*	PH01001487	A	P	A	A	P	P	P	A	A	Seq
*Brachypodium distachyon*	Bradi3g12627	A	A	A	A	P	A	P	A	A	Seq
*Brachypodium stacei*	Brast03G311000	A	A	A	A	P	A	P	A	A	Seq
*Hordeum vulgare*	MLOC_5438	A	A	A	A	P	P	P	A	A	Seq
*Aegilops tauschii*	F775_52675	A	A	A	A	P	P	P	A	A	Seq
*Triticum urartu*	TRIUR3_22724	A	A	A	ND	P	P	P	A	A	Seq
*Eragrostis tef*	JN672670	A	A	A	A	A	P	P	A	A	Seq
*Eragrostis tef*	JN672669	A	A	A	A	A	P	P	A	A	Seq
*Zoysia* spp.	Not assigned	A	A	A	A	A	A	P	A	A	LR-PCR/Seq
*Oropetium thomaeum*	Oropetium_20150105_14444	A	P	A	A	A	A	P	A	A	Seq
*Eleusine coracana*	Not assigned	A	P	A	A	A	A	P	A	A	Seq
*Micraira subilifolia*	Not assigned	A*	A*	A*	A*	P	P	ND	A	A	PCR/Seq
*Phragmites australis*	Not assigned	A*	P*	A*	A*	P	P	ND	A	A	PCR/Seq
*Danthoniopsis dinteri*	Not assigned	A	P	A	A	P	ND	ND	A	A	PCR
*Chasmanthium laxum*	Not assigned	A	P	A	A	P	P	P	A	A	Seq
*Paspalum vaginatum*	Not assigned	A	P	A	A	A	P	P	A	A	LRPCR/Seq
*Zea mays*	GRMZM2G315375	A	P	A	A	P	P	P	A	A	Seq
*Sorghum bicolor*	AY372819.1	A	P	A	A	P	P	P	A	A	Seq
*Arundinella hirta*	Not assigned	A	P	A	A	P	ND	P*	A	A	PCR/Seq
*Andropogon virginicus*	Not assigned	A	P	A	A	P	P	P	A	A	Seq
*Andropogon gerardii*	Not assigned	A	P	A	A	P	ND	P*	A	A	PCR/Seq
*Acroceras macrum*	Not assigned	A	P	A	A	A	ND	P*	A	ND	PCR/Seq
*Sacciolepis myosuroides*	Not assigned	A	P	A	A	P	ND	P*	A	A	PCR/Seq
*Dichanthelium* *oligosanthes*	Not assigned	A	P	A	A	A	P	P	A	A	Seq
*Panicum virgatum*	Pavir.6NG364900	A	P	A	A	A	P	P	A	A	Seq
*Panicum virgatum*	Pavir.6KG414900	A	P	A	A	A	P	P	A	A	Seq
*Panicum hallii*	Pahal.F00317	A	P	A	A	A	P	P	A	A	Seq
*Setaria italica*	Seita.6G253500	A	A	A	A	A	P	P	A	A	Seq
*Setaria viridis*	Sevir.6G258000	A	A	A	A	A	P	P	A	A	Seq
*Cenchrus americanus*	CaABCB1	A	A	A	A	A	P	P	A	A	Seq
*Brachiaria* spp.	Not assigned	A	A	A	A	A	P	ND	A	ND	PCR
*Urochloa abyss*	Urochola_abyss_gt1kb	A	A	A	A	A	P	P	A	A	Seq
Basal angiosperm											
*Amborella trichopoda*	evm_27.TU.AmTr_v1.0_scaffold00076.86	P	P	P	P	P	P	P	P	P	Seq

^a^ *Seq*: Sequence information from cloned genes or whole-genome sequencing; *PCR*: PCR amplification across two introns (1 + 2, 3 + 4) or single introns; *PCR/Seq*: PCR amplification across introns followed by sequencing of some of the amplicons (Sanger sequenced amplicons are indicated with an asterisk (*)); *LR-PCR/Seq*: Long-range PCR across all introns followed by sequencing of the amplification product

^b^ *P* present, *A* absent; ND indicates introns for which no data is available on intron status

Because *ABCB1* is a member of a multigene family, we also examined the structure of *ABCB19*, the closest extant paralog of *ABCB1*. *ABCB1* and *ABCB19* have partially overlapping functions in *Arabidopsis* as both copies need to be knocked-out to see a phenotype [47]. In grasses, however, inactivation of *ABCB1* is sufficient to obtain a dwarf phenotype [23, 24]. *ABCB19* contained nine introns in all sequenced dicot species, except the two *Brassica* species analyzed (Additional file 5). *ABCB19* is duplicated in the *Brassica* lineage and both gene copies (Brara.F03123; Brara.I00328) lacked intron 7 in *B. rapa* and one copy (Bo9g008680) lacked intron 7 in *B. oleracea* (Additional file 1: Table S2). If intron loss occurred before the duplication of *ABCB19*, one of the *ABCB1* copies must have gained an intron in *B. oleraceae*. Alternatively, two independent losses occurred of intron 7, one in one of the duplicated *ABCB19* copies in the lineage leading to and before the divergence of *B. rapa* and *B. oleraceae*, and one in the other duplicated gene copy in the *B. rapa* lineage. *ABCB19* also underwent a duplication event before the radiation of the grasses. The chromosomal location of the *ABCB19* copies in grasses indicated that this event was different from the pre-grass ancestral whole-genome duplication [48, 49]. In all grass species, one *ABCB19* copy contained nine introns while the second copy lacked intron 3, indicating that the loss of intron 3 likely occurred soon after *ABCB19* was duplicated (Additional file 6). In the non-grass monocot *Zostera marina*, *ABCB19* was present in one copy and lacked introns 5, 6, 7 and 8. *ABCB19* was incorrectly annotated as three gene models (Zosma85g00160, Zosma85g00150 and Zosma85g00140) in *Z. marina* due to the presence of a stop codon in exon 2. The other non-grass monocots analyzed contained one or two copies of *ABCB19* with nine introns each (Additional file 1: Table S2; Additional file 6).

All intron positions were conserved between *ABCB1* and *ABCB19* except for the position of intron 8 (Additional file 7). *ABCB1* intron 8 was located ~500 bp upstream of *ABCB19* intron 8. To avoid confusion, *ABCB1* intron 8 is hereafter referred to as intron 8-ABCB1 and *ABCB19* intron 8 as intron 8-ABCB19 (Table 4). A comparison of *ABCB1* and *ABCB19* with other close *ABCB* family members in *Arabidopsis* (*ABCB2*: AT4G25960; *ABCB10*: AT1G10680; *ABCB13*: AT1G27940; *ABCB14*: AT1G28010) and rice (LOC_Os02g46680, LOC_OS08g05690, LOC_Os08g05710) [50] showed that intron8-ABCB1 was present in the highly divergent linker region connecting NBD1 and TMD2 (Additional file 8). Linker regions are targets of protein-kinase phosphorylation which alters ABCB activity [51]. The rice *ABCB* homologs LOC_Os08g05690 and LOC_Os08g05710, and *ABCB1* and *ABCB19* in the basal angiosperm *Amborella* also carried an intron in that region. Intron8-ABCB19 was present in all *ABCB* genes analyzed, except *ABCB1* and the rice *ABCB* ortholog (LOC_Os02g46680) most closely related to *ABCB2/10* [50] (Additional file 1: Table S3). *ABCB1* in *Amborella* also lacked intron8-ABCB19. The presence of both intron8-ABCB1 and intron8-ABCB19 in *ABCB19* in *Amborella* suggests that presence is the ancestral state for both introns in *ABCB* genes. *Arabidopsis ABCB2* and *ABCB10* carried three additional introns after the 9th intron (introns 10, 11 and 12). Introns 11 and 12 were also present in rice *ABCB2/10*, but intron 12 was absent from rice *ABCB2/10*. *Amborella ABCB19* carried an additional intron, designated Amborella-intron10, which differed in its position from intron 10 in *ABCB2/10.* This intron was absent in all dicot and monocot *ABCB* genes analyzed and may represent an intron gain in *Amborella* (Additional file 1: Table S3). Assuming presence was the ancestral state for all other introns, the ancestral *ABCB* gene had at least 14 exons and 13 introns (Additional file 1: Table S3). Introns 10, 11 and 12 were likely lost before the divergence of *ABCB1* and *ABCB19*, while intron8-ABCB19 and intron8-ABCB1 were lost from *ABCB1* and *ABCB19*, respectively, shortly after the divergence of these two genes from their common ancestor.

**Table 4:** Comparative gene structure of *ABCB1* and *ABCB19* in *Arabidopsis* and rice

			Intron number									
Species	Locus ID	Gene	1	2	3	4	5	6	7	8-ABCB1	8-ABCB19	9
			Intron presence/absence^a^									
*O. sativa*	LOC_Os08g45030	*ABCB1*	A	A	A	A	P	A	P	A	A	A
*O. sativa*	LOC_Os04g38570	*ABCB19*	P	P	P	P	P	P	P	A	P	P
*A. thaliana*	AT2G36910	*ABCB1*	P	P	P	P	P	P	P	P	A	P
*A. thaliana*	AT3G28860	*ABCB19*	P	P	P	P	P	P	P	A	P	P

^a^ *P* present, *A* absent

### PCR analysis of intron presence in *ABCB1*

Intron presence in 19 species for which no genomic sequence was available was analyzed by PCR (Table 3). *Cenchrus americanus*, *O. sativa* and *Z. mays*, three species for which the *ABCB1* gene structure was known, were used as controls. For most introns, a single amplification product was obtained in the analyzed species, allowing unambiguous determination of the presence/absence of that intron. Some primer pairs, however, generated two amplicons of different sizes with one fragment corresponding in size to an intron-less amplicon and the other to an amplicon containing an intron. This was observed especially, although not exclusively, in species outside the *Poaceae*. Multiple fragments might have been due to allopolyploidy of the analyzed species (the ploidy level of many non-sequenced species was unknown) whereby different subgenomes carried *ABCB1* genes with a different intron composition. However, especially for the non-grass species, multiple amplicons might have been due to non-specific amplification of other ABC genes. As the identity of these amplicons was not confirmed by sequencing, we conservatively scored intron presence/absence in species that displayed two PCR fragments as ‘not determined’ (ND) (Table 3). Exon 2 and exon 4 were small, and because introns 1, 3 and 4 were absent in all sequenced grasses initially analyzed, primers were designed to simultaneously amplify introns 1 and 2, and introns 3 and 4. Fragments of varying sizes were obtained and the sizes of the amplification products were used as guidance to determine the presence/absence of the individual introns. For introns 1 and 2, amplicons with a size similar to those of rice and pearl millet were considered to lack both introns 1 and 2, and amplicons with a size similar to that in maize were considered to lack intron 1 but carry intron 2. Initially, we assumed that amplicons with sizes larger than that obtained in maize carried both introns 1 and 2. However, amplification with primer ABCB1_F8 (Table 1) which spanned the exon 1 – exon 2 boundary and hence should anneal only if intron 1 was absent, suggested that several of the larger fragments likely lacked intron 1 and potentially carried a larger intron 2. The presence/absence scores for introns 1 and 2 in Table 3 reflect the latter interpretation. For introns 3 and 4, small, intermediate and larger amplicons were obtained. Small fragments lacked both introns 3 and 4 (e.g. rice, pearl millet and maize), larger fragments were predicted to contain both introns 3 and 4, while intermediate fragments were scored as carrying only intron 3 or intron 4. Because introns 5 to 9 were amplified individually, the presence/absence of these introns could be determined unambiguously. Amplification across intron 7, which is positioned in an ATP-binding cassette transporter nucleotide binding domain, was complicated by the high homology of this region across *ABC* gene family members. Intron presence in all intron 7 amplicons and in some other amplicons was confirmed by Sanger sequencing (Table 3; Additional file 9).

### Validation of the PCR-predicted gene structure by sequence analysis

Long-range amplification across all *ABCB1* introns was achieved for *Typha*, *Ecdieocolea*, *Streptochaeta*, *Mayaca, Paspalum vaginatum, Zoysia* and *Phyllostachys* and the amplification products sequenced (Genbank IDs KY939582–939588; Sequence alignments are given in Additional file 9). The intron composition of these *ABCB1* orthologs is given in Table 3. Sequencing confirmed the results of the PCR analyses across introns for the grass species *P. vaginatum, Zoysia*, *Phyllostachys,* and *Streptochaeta*. In *Mayaca* and *Ecdeiocolea*, the size of the PCR fragment amplified across introns 3 + 4 suggested the presence of either intron 3 or intron 4. Sequencing confirmed that intron 3 was present and intron 4 was absent in *Mayaca* but also showed that both introns were present in *Ecdeiocolea*. Inspection of this region showed that introns 3 and 4 in *Ecdeiocolea ABCB1* had a combined size of 140 bp while the size of intron 3 in *Mayaca* was 97 bp. In comparison, the combined size of introns 3 and 4 in *Streptochaeta ABCB1* was 208 bp. The intermediate size of the intron 3 + 4 amplicon in *Ecdeiocolea* had been interpreted as the presence of a single intron but, in fact, this amplicon comprised two smaller introns (Additional file 9). The other species with an intermediate intron 3 + 4 fragment size, *Anomochloa,* was confirmed by sequencing to carry intron 3 and lack intron 4. Overall, we observed variation for the presence of all introns except intron 7 across the sample of tested monocot species (Table 3).

### Conserved motifs in intron 7

With a width setting of 12 letters, four motifs were identified by MEME in intron 7 with e-values <1.0e^−10^ (Additional file 10). The most common motif (e-value 1.4e^−90^) had a highly conserved core motif of 8 bp (GTAACATG) that was present without mismatches in 44 angiosperms (55 *ABCB1* genes), and with 1 and 2 bp mismatches in an additional 13 angiosperms (15 genes) and six angiosperms (seven genes), respectively (Additional file 1: Table 4). We defined a core motif as consisting of bases with position-specific probabilities >80%). Three *ABCB1* genes had three mismatches, but all three were duplicated copies with the other copy carrying the motif with a maximum of two mismatches. Only one species, the monocot *Zostera marina* (Zosma16g00300) contained four mismatches in the 8 bp motif suggesting that the motif may have been eliminated in this species. The size of intron 7 in *Z. marina* is 68 bp, the smallest size for intron 7 across the analyzed species. A second motif (TTTKGTCARSAA; e-value 1.5e^-86^) was highly conserved (0, 1 or 2 mismatches of the nine core bases) in 76 *ABCB1* genes (57 angiosperms) (Additional file 1: Table S5). The two motifs are 2 bp apart in *Arabidopsis* but 378 bp apart in rice, suggesting they are separate motifs. Neither of these motifs were found in the databases checked (FootprintDB (*floresta.eead.csic.es/footprintdb*); Plant Cistrome Database (*neomorph.salk.edu/dev/pages/shhuang/dap_web/pages/index.php*); Plant DHS (*www.plantdhs.org*); miRBase (*www.mirbase.org*)).

To determine the probability of finding motifs in sequences similar in length to *ABCB1* intron 7, we conducted MEME analyses on six concatenated *ABCB19* introns (median length 1073 bp), on *ABCB19* intron 9 (median length 1266 bp), and on *ABCB1* intron 7 (median length 1084 bp) across 59 species. Motif ‘GTAACATG’ was the top motif identified in intron 7 with an e-value of 5.7e^−64^ followed by ‘TTTKGTCARSAA’ with an e-value of 1.6e^−55^_._ The most significant motif identified in intron 9 in *ABCB19* was ‘GCAYGTGCTTYC’ (6.9e^−61^) which comprises a G-box motif (‘CACGTG’) [52]. In the concatenated *ABCB19* introns, the top motif was TYAGATCYMA’ (2.2e^−47^). This motif was largely found in intron 1 in dicots (83% of dicots) and in intron 4 in monocots (75% of monocots). ‘AGATCCAA’ (AG-motif) has been identified as a *cis*-regulatory motif in the promoter region of *NtMyb2* involved in the response to wounding and elicitor treatment [53]. We also analyzed random sequence, either upstream regions of genes or randomly selected regions within genes, of similar length to *ABCB1* intron 7 for the presence of motifs. For both datasets, all motifs identified in 100 MEME analyses in six monocots and four dicots had e-values higher than the e-value of the ‘GTAACATG’ intron 7 motif across the same 10 species (*p*-value = 0) (Additional file 1: Tables S6 and S7). For motif ‘TTTKGTCARSAA’, two out of the 100 MEME analyses for random upstream sequence (*p*-value = 0.02) and zero out of 100 for random genic sequence (*p*-value = 0) had e-values equal to or lower than the target motif. We can thus reject the null hypothesis at alpha level of 0.05 that these motifs were found by chance.

### Transcript levels of *ABCB1* in *Mimulus guttatus*

**Fig. 2:**
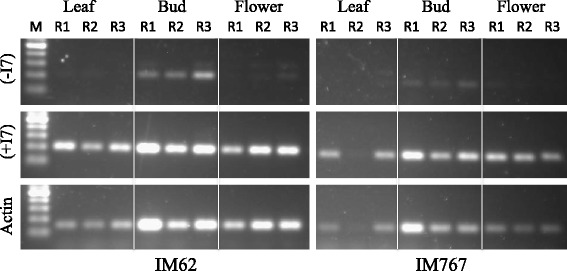
Agarose gel showing amplicons obtained by semiquantitative PCR using cDNA from leaves, buds and flowers from *M. guttatus* accessions IM62 and IM767. PCR was done on three biological replicates (R1, R2 and R3). (−I7) indicates amplification products obtained with primers designed against *ABCB1* gene copy Migut.L01707, which lacks intron 7. (+I7) indicates amplification products obtained with primers designed against *ABCB1* gene copy Migut.J00652, which carries intron 7. Actin was used as control

Transcript levels of Migut.J00652 were significantly higher than those of Migut.L01707 in all three organs in both inbred lines tested (Fig. 2). To ensure that the higher amplicon levels in Migut.J00652 compared to Migut.L01707 were not caused by differences in amplification efficiency between the two primer sets and/or genes, the primers were also tested on genomic DNA. Similar levels of amplification were obtained in Migut.J00652 and Migut.L01707 (Additional file 11).

## Discussion

### The genomic structure of *ABCB1* but not *ABCB19* is highly dynamic

Structural data from a range of sequenced dicot and monocot genomes (Table 3) suggests that the ancestral *ABCB1* gene consisted of 10 exons and nine introns. In the discussion below, we will refer to introns by their ancestral intron number. The ancestral gene structure has been maintained in the majority of dicot species and about half of the non-grass monocots analyzed. In most grass species, however, the maximum number of introns in *ABCB1* was four. Although our analysis focused on only a single gene, *ABCB1*, a number of observations could be made. Firstly, the overall frequency of intron loss events in *ABCB1* was less than 1% in dicots and 6% in monocots when we assume conservatively that neighboring introns were lost together and that losses in related species occurred in a common ancestor. The total number of *ABCB1* introns lost in the monocots was close to 50%. Hence, intron loss was not random across the phylogenetic tree. Secondly, intron removal was precise in 100% of the cases for which exon-intron boundary sequence was available. Thirdly, in both dicots and monocots, all *ABCB1* introns except intron 5 were flanked by two to four bases of microhomology (Additional files 3 and 4). However, in grasses, the penultimate base of exon 2 and exon 6 was significantly different in *ABCB1* genes in which the downstream intron was lost compared to genes that carried the downstream intron (*p*-value <0.001). Introns 2 and 6 were phase I introns, and hence the second to last base of the upstream exon was the third base in a codon. Following intron loss, these bases no longer played a role in splicing, and hence were free to mutate provided amino acid identity was maintained [16]. Fourthly, many of the loss events in *ABCB1* involved non-adjacent introns. Irrespective of whether intron removal occurred through recombination with a cDNA or NHEJ, most genes underwent multiple independent loss events. Finally, intron loss, including parallel intron loss, occurred more frequently than previously thought. An initial analysis of *ABCB1* in *Arabidopsis*, *Musa*, rice, *Brachypodium*, pearl millet, foxtail millet, sorghum and maize had suggested that introns 1, 3, 4, 8 and 9 had been lost before the radiation of the grasses, intron 5 had been lost in the Paniceae lineage, intron 6 in the Pooideae lineage and intron 2 had been lost independently in both the Paniceae and Pooideae. However, as more genomes were added to the analysis, many additional intron loss events were observed. Intron 2 was lost independently at least eight times in angiosperms with seven of the events being in monocots (Fig. 1). The most recent loss of intron 2 occurred in the Melinidinae/Cenchrinae lineage (represented in our study by *Cenchrus americanus*, *Setaria spp.*, *Brachiaria spp.* and *Urochloa abyss*) after it split from the Panicinae (represented by *Panicum virgatum* and *Panicum hallii*) sometime during the past 13.1 MYA [54, 55]. Intron 5 was lost independently at least five times, with at least three losses having taken place within the grass sub-family Panicoidae, and intron 6 was lost at least three times in the grass family. Introns 4 and 8 were each lost a total of three times in the analyzed angiosperms, and intron 9 was lost a total of five times. Intron 1 was lost once in the common ancestor of the grasses and the *Ecdeiocolea* lineage. Considering the highly dynamic nature of the genomic structure of *ABCB1* in the sample of analyzed species, further expansion of the species set will likely identify additional events of intron loss. Interestingly, the genomic structure of *ABCB19*, which also has nine introns and, in *Arabidopsis*, is functionally redundant to *ABCB1* [47] has largely remained stable over more than 100 million years of evolution.

### Potential causes of intron recalcitrance to loss

It is unclear what caused the high frequency of intron loss in *ABCB1*. Some studies have shown intron loss to be correlated with gene duplications [10, 56, 57]. *ABCB1,* however, is single copy in many of the species that underwent intron loss. It has also been shown that smaller introns have a higher tendency to undergo evolutionary loss than larger introns [9, 11, 58, 59]. In the monocot *ABCB1* genes, where we found the majority of intron loss events, introns 1 to 5, 8-ABCB1 and 9 have mean and median lengths of <100 bp (Additional file 1: Table S8). This intron size is similar to that found by Wang et al. [9] for lost introns. Intron 6, which was lost independently at least three times, has mean and median lengths of 351 bp and 369 bp, respectively. However, the length of intron 6 in the Chloridoideae, Ehrhartoideae and Pooideae, the three grass subfamilies in which loss of intron 6 was observed, is at most 101 bp. This suggests that a reduction in intron size likely preceded the phenomenon of evolutionary intron removal, at least in the case of intron 6 in *ABCB1*. In contrast, the conserved intron 7 had mean and median lengths of 1030 bp and 1071 bp, respectively (Additional file 1: Table S8). In monocot *ABCB19*, where intron composition was largely maintained across species, introns 3, 6 and 8-ABCB19 had mean and median lengths of <100 bp, and introns 1, 5 and 7 were <200 bp. Intron 2 (mean length: 300 bp; median length: 374 bp), intron 4 (mean length: 1035 bp; median length: 978 bp) and intron 9 (mean length: 744 bp; median length: 505 bp) were larger (Additional file 1: Table S9). While there was a trend for shorter introns to be lost more frequently in *ABCB1* in the grasses, introns of comparable size in dicot *ABCB1* and in *ABCB19* were retained, suggesting that intron size was likely not the only factor driving intron loss in *ABCB1*. It is possible that some introns were retained, regardless of their size, because they carried functionally significant motifs. For example, the regulatory motif ‘AGATCCAA’ was identified in intron 1 in dicots and intron 4 in monocots in *ABCB19*. The G-box motif ‘CACGTG’ was identified in intron 9 in *ABCB19*.

Two highly conserved motifs were also identified in the large intron 7 in *ABCB1* with e-values lower or similar to those of the G-box and AG-motifs identified in *ABCB19*. Furthermore, analysis of random sequence in 10 species across the monocot-dicot divide showed that the probability of identifying these motifs by chance was less than 5%. We therefore hypothesize that these motifs are functional. The motifs do not correspond to any previously reported motifs in plant species as far as we could establish. It is interesting to note that the marine *Z. marina* was the only species in which intron 7 did not carry these motifs. *ABCB1* is involved in basipetal auxin transport, which regulates the formation of procambium and vascular cambium [60]. These cell types give rise to the vascular tissues, which are responsible for long-distance transport and provide support to the plant. *Zostera marina* has a functional vascular system, but water transport is greatly reduced [61]. Furthermore, as an aquatic plant, *Z. marina* does not require the same level of stem support as a terrestrial plant. It would be interesting to test whether the absence of the motifs affected the level of *ABCB1* in *Z. marina* and whether this species has reduced basipetal auxin transport compared to terrestrial land plants. Because *Mimulus guttatus* carried two *ABCB1* gene copies, one with intron 7 and one without intron 7, we examined the transcript levels of the two gene copies. Interestingly, we found that the gene copy with intron 7 had significantly higher transcript levels than the gene copy without intron 7. While these results are potentially exciting, it is possible that the observed expression differences are effected by genetic variation between the two gene copies other than the differential presence of intron 7. We have initiated experiments to test the function of intron 7 using intron deletion and motif knock-out experiments in *Arabidopsis*. Analysis of the presence of these motifs in other orthologous angiosperm introns is also in process to gain insights into their potential role in gene regulation.

We also considered whether chromosomal location could play a role in intron loss. Recombination typically increases from the centromere to the telomere and, in grasses, this gradient can be very steep [62–64]. Distally located genes could be more prone to undergoing double strand breaks [65] which could potentially be repaired using homologous recombination with the corresponding cDNA or by NHEJ. In rice, the grass species with a chromosome structure that best approximates that of the grass ancestral genome [66, 67], *ABCB1* is located in the most distal 200 kb of chromosome 8. In contrast, *ABCB19* is located more than 12.5 Mb proximal to the telomere on rice chromosome 4. Wang and colleagues [9] examined the distribution of genes that underwent intron loss along the chromosome and did not find a significant correlation between intron loss and chromosomal position. However, chromosomal positions were determined in extant species, many of which have undergone rearrangements since their divergence from a common ancestor [67]*.* Such an analysis should therefore be limited to those genes for which the species or lineage in which an intron was lost can be identified and the chromosomal organization of the species/lineage that underwent intron loss is known or can be determined.

### Intron loss or gain?

Throughout our analysis, we have assumed that differential presence of introns in *ABCB1* was caused by intron loss. Although intron loss has been shown to occur more frequently than intron gain [9], a valid concern is that some of the parallel intron loss events described for *ABCB1* could, in fact, represent single loss events that occurred early in evolution and were followed by intron gain in specific lineages. Using known phylogenetic relationships between the species [54], we determined which model – one consisting solely of intron losses (Fig. 1) or one that combined intron loss with gain (Additional file 12) – required the least number of events to explain intron evolution. This analysis was limited to the grass clade because phylogenetic relationships are well established in this family, and to introns 2, 5 and 6 because these introns were differentially present in several grass lineages. Using a maximum parsimony approach, a minimum of three events were required to explain the presence/absence variation of intron 6 by intron loss alone but a total of four events (two losses and two gains) were required to explain the intron composition by a mixed model. In this particular case, the intron loss model is more likely than a mixed model of intron loss and gain. For intron 5 and intron 2, both intron loss and mixed models are equally likely as the intron presence/absence variation can be explained by four loss events, or three loss and one gain events. If an intron is gained independently, then the sequence of this gained intron would likely be unique. However, because introns are typically not under selective constraint, they evolve fast [68]. Therefore, significant intron homology can only be identified between species that are closely related. We currently do not have sequence information of closely related species that differ by the presence/absence of an intron to test if intron gain truly occurs.

## Conclusions

The *ABCB1* gene underwent dynamic structural changes in the angiosperms, particularly in the order Poales, while the structure of *ABCB19* largely remained constant. This is the first gene in which genomic intron loss at that scale has been reported. Precise removal of introns, preferential removal of smaller introns and the presence of two to four bases of microhomology suggested that NHEJ was the predominant mechanism leading to intron loss. Apparent loss of microhomology in 5′ exons of phase I introns that had been removed during evolution could be explained by relaxation of the selective constraint imposed by the splicing machinery on these bases.. However, the cause for the high rate of intron removal in *ABCB1* remains unclear. An analysis of the location of genes along the centromere-telomere recombinational gradient at the time of intron loss is needed to determine whether intron loss is correlated with recombination rates. The identification of two highly conserved motifs in intron 7 and the differential accumulation of *ABCB1* transcripts in two *M. guttatus* gene copies that differ by the presence of intron 7, could indicate that these unknown motifs play a role in the function of *ABCB1*.

## Additional files

Additional file 1: **Table S1.** *ABCB1* and *ABCB19* sequences analyzed and their source. **Table S2.** Presence/absence polymorphism of introns in *ABCB19* in sequenced angiosperms. **Table S3.** Presence/absence polymorphisms of introns in *ABCB* genes in *Arabidopsis* and rice, and of *ABCB1* and *ABCB19* in *Amborella trichopoda.* **Table S4.** Location in intron 7 of motif GTAACATG and number of mismatches present in the motif in 81 angiosperms. **Table S5.** Location in intron 7 of motif TTTKGTCARSAA and number of mismatches present in the motif in 81 angiosperms. **Table S6.** Two top motifs identified by MEME in *ABCB1* intron 7 and top motif identified in 1080 bp of sequence upstream of randomly selected genes across six monocots and four dicots, and their corresponding e-value. The analysis was repeated 100 times. **Table S7.** Two top motif identified by MEME in *ABCB1* intron 7 and top motif identified in 1080 bp of sequence downstream of a random starting point in randomly selected genes across six monocots and four dicots, and their corresponding e-value. The analysis was repeated 100 times. **Table S8.** Size of introns of *ABCB1* across monocots. **Table S9.** Size of introns of *ABCB19* across monocots. (XLSX 112 kb)

Additional file 2: Maximum likelihood protein tree of analyzed ABCB1 (in blue) and ABCB19 (in red) sequences. The maximum likelihood tree was generated by RAxML from alignments of analyzed ABCB1 and ABCB19 proteins. Protein names are as listed in Additional file 1: Table S1. ABCB1 and ABCB19 proteins are color-coded in blue and red, respectively. (PDF 394 kb)

Additional file 3: Multiple sequence alignment of monocot *ABCB1* genes**.** A multiple sequence alignment was generated for genomic and, for a few species, cDNA sequences of monocot *ABCB1* genes obtained from available whole genome sequence data or sequenced *ABCB1* genes. Differentially colored regions indicate exons and introns. (PDF 962 kb)

Additional file 4: Multiple sequence alignment of dicot *ABCB1* genes. A multiple sequence alignment was generated for genomic and, for a few species, cDNA sequences of dicot *ABCB1* genes obtained from available whole genome sequence data or sequenced *ABCB1* genes. Differentially colored regions indicate exons and introns. (PDF 1331 kb)

Additional file 5: Multiple sequence alignment of dicot *ABCB19* genes. A multiple sequence alignment was generated for genomic and, for a few species, cDNA sequences of dicot *ABCB19* genes obtained from available whole genome sequence data or sequenced *ABCB19* genes. Differentially colored regions indicate exons and introns. (PDF 1643 kb)

Additional file 6: Multiple sequence alignment of monocot *ABCB19* genes. A multiple sequence alignment was generated for genomic and, for a few species, cDNA sequences of monocot *ABCB19* genes obtained from available whole genome sequence data or sequenced *ABCB19* genes. Differentially colored regions indicate exons and introns. (PDF 1568 kb)

Additional file 7: Multiple sequence alignment of genomic and cDNA sequences of *ABCB1* and *ABCB19* from *Arabidopsis* and rice. The multiple sequence alignment of genomic and cDNA sequences of *ABCB1* and *ABCB19* from *Arabidopsis* and rice provides information on the comparative location of introns in *ABCB1* and *ABCB19*. Differentially colored regions indicate exons and introns. (PDF 193 kb)

Additional file 8: Multiple sequence alignment of selected ABCB proteins from *Arabidopsis* and rice, and of ABCB1 and ABCB19 from *Amborella*. A multiple sequence alignment was generated of protein sequences of selected *ABCB* family members from *Arabidopsis* and rice, and of ABCB1 and ABCB19 from *Amborella*. The positions of intron 8-ABCB1 in the variable linker region (~amino acids 681–762) in *Arabidopsis* and *Amborella* ABCB1, *Amborella* ABCB19, and rice ABCB homologs LOC_Os08g05690 and LOC_Os08g05710 are indicated by a black box around the relevant amino acid. (PDF 119 kb)

Additional file 9: Multiple sequence alignment of genomic sequences from regions of *ABCB1* obtained by long-range PCR across all nine introns or amplification across one or a small number of introns. (PDF 337 kb)

Additional file 10: MEME output of the motifs discovered in intron 7 in 81 *ABCB1* orthologs from 27 monocots, 32 dicots and the basal angiosperm *Amborella trichopoda*. Four sequence motifs were identified by MEME in intron 7 at an e-value threshold of 1.0e^-10^. (PPTX 70 kb)

Additional file 11 Agarose gel showing amplification products obtained in *Mimulus guttatus* accessions IM62 and IM767 using primers designed against *ABCB1* gene copies Migut.L01707 and Migut.J00652. Similar amplicon levels were obtained from genomic DNA for Migut.L01707 and Migut.J00652 in both accessions, indicating that both primers/gene regions have similar amplification efficiencies. (PPTX 2838 kb)

Additional file 12: Mixed model for *ABCB1* intron variance in the Poales explained by both intron loss and intron gain events. Intron loss events are shown by red stars and a minus sign (−) followed by the intron that was lost. Intron gain events are shown by green stars and a plus sign (+) followed by the intron that was gained. Members of the grass family are represented in black while non-grass monocots are in blue. The vertical bars indicate different grass subfamilies. (PDF 922 kb)

## Abbreviations

ABCB1: ATP Binding Cassette Subfamily B Member 1
DSB: Double Strand Break
MEME: Multiple EM for Motif Elicitation
MSA: Multiple Sequence Alignment
NBD: Nucleotide Binding Domain
NHEJ: Non-Homologous End Joining
RT: Reverse Transcription
TMD: Transmembrane Domain
UGA: University of Georgia
