# Supplementary figures and images for: Parallel loss of introns in the ABCB1 gene in angiosperms
Source: BMC Evol Biol. 2017 Dec 4;17:238. doi: 10.1186/s12862-017-1077-x (PMC5716013; doi:10.1186/s12862-017-1077-x)

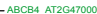

Supplement: Supplementary file 2 — Maximum likelihood protein tree of analyzed ABCB1 (in blue) and ABCB19 (in red) sequences. The maximum likelihood tree was generated by RAxML from alignments of analyzed ABCB1 and ABCB19 proteins. Protein names are as listed in Additional file 1: Table S1. ABCB1 and ABCB19 proteins are color-coded in blue and red, respectively. (PDF 394 kb) [file 12862_2017_1077_MOESM2_ESM.pdf]

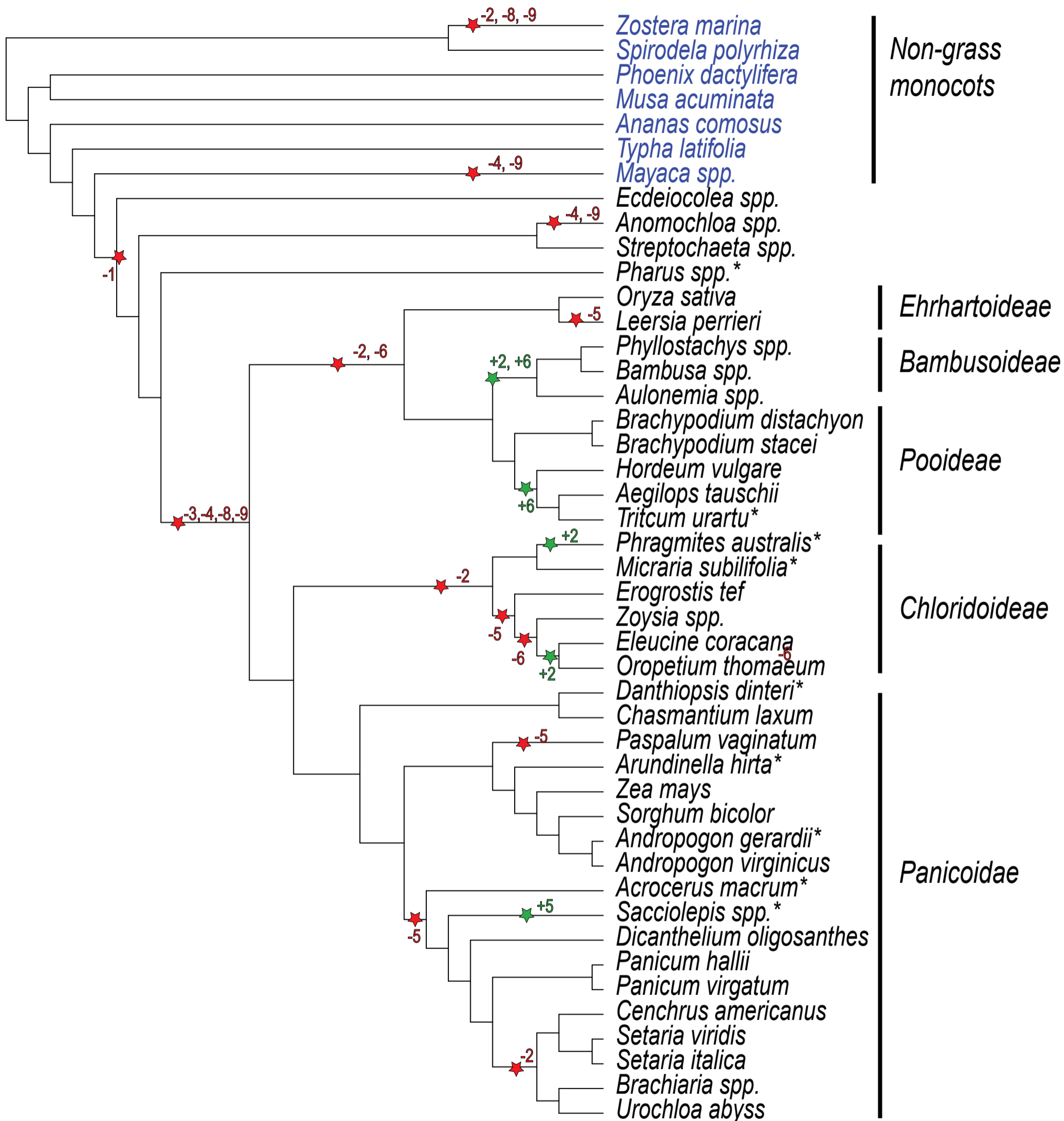

Supplement: Supplementary file 12 — Mixed model for ABCB1 intron variance in the Poales explained by both intron loss and intron gain events. Intron loss events are shown by red stars and a minus sign (−) followed by the intron that was lost. Intron gain events are shown by green stars and a plus sign (+) followed by the intron that was gained. Members of the grass family are represented in black while non-grass monocots are in blue. The vertical bars indicate different grass subfamilies. (PDF 922 kb) [file 12862_2017_1077_MOESM12_ESM.pdf]
