# Supplementary material for: Parallel loss of introns in the ABCB1 gene in angiosperms
Source: BMC Evol Biol. 2017 Dec 4;17:238. doi: 10.1186/s12862-017-1077-x (PMC5716013; doi:10.1186/s12862-017-1077-x)
Supplement: Supplementary file 10 — MEME output of the motifs discovered in intron 7 in 81 ABCB1 orthologs from 27 monocots, 32 dicots and the basal angiosperm Amborella trichopoda. Four sequence motifs were identified by MEME in intron 7 at an e-value threshold of 1.0e-10. (PPTX 70 kb) [file 12862_2017_1077_MOESM10_ESM.pptx]

## Slide 1
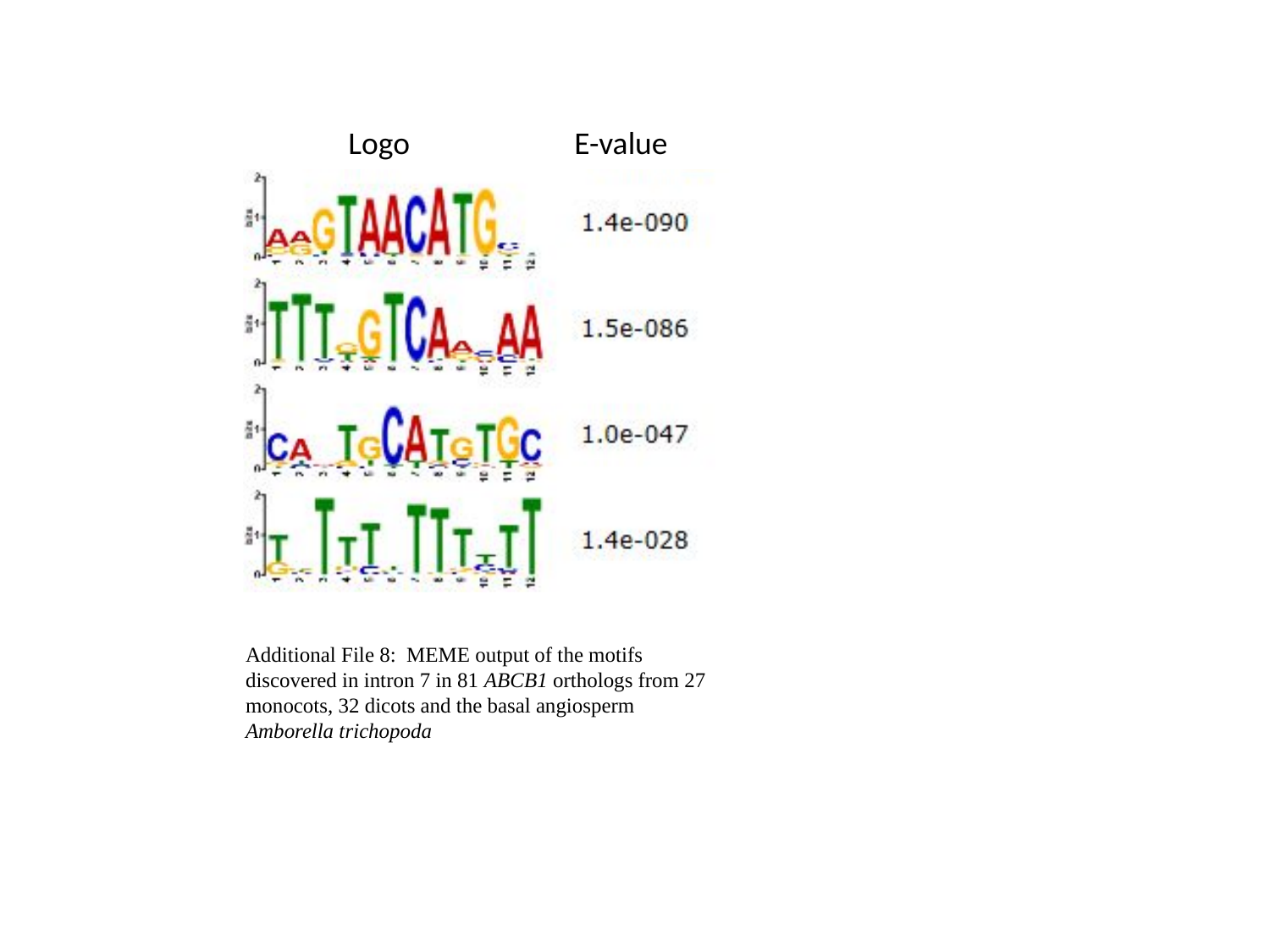

Logo
E-value
Additional File 8: MEME output of the motifs discovered in intron 7 in 81 ABCB1 orthologs from 27 monocots, 32 dicots and the basal angiosperm Amborella trichopoda
