# Supplementary material for: Parallel loss of introns in the ABCB1 gene in angiosperms
Source: BMC Evol Biol. 2017 Dec 4;17:238. doi: 10.1186/s12862-017-1077-x (PMC5716013; doi:10.1186/s12862-017-1077-x)
Supplement: Supplementary file 11 — Agarose gel showing amplification products obtained in Mimulus guttatus accessions IM62 and IM767 using primers designed against ABCB1 gene copies Migut.L01707 and Migut.J00652. Similar amplicon levels were obtained from genomic DNA for Migut.L01707 and Migut.J00652 in both accessions, indicating that both primers/gene regions have similar amplification efficiencies. (PPTX 2838 kb) [file 12862_2017_1077_MOESM11_ESM.pptx]

## Slide 1
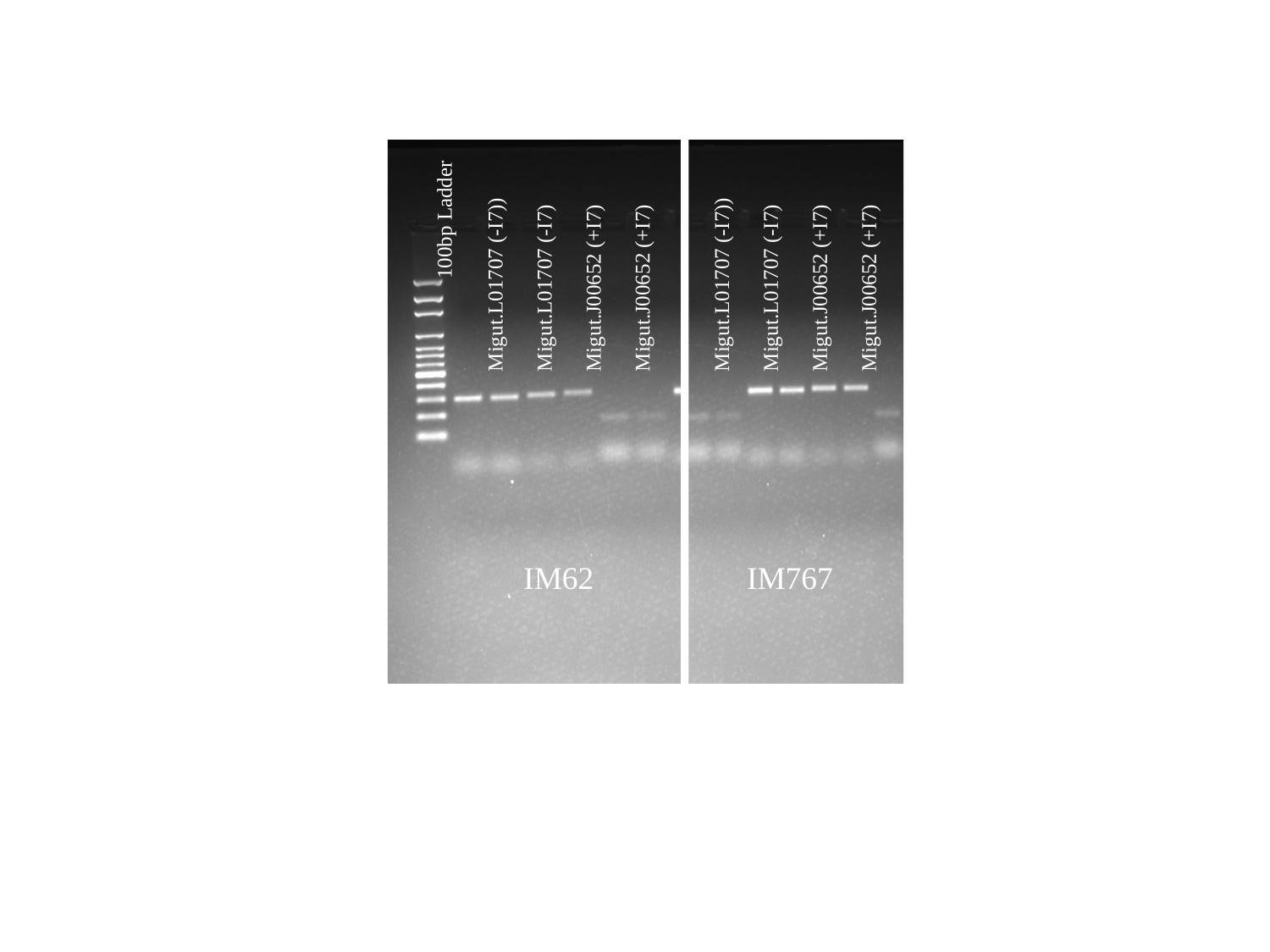

100bp Ladder
Migut.L01707 (-I7)
Migut.L01707 (-I7)
Migut.L01707 (-I7))
Migut.J00652 (+I7)
Migut.J00652 (+I7)
Actin rep1
Migut.L01707 (-I7))
Actin rep2
Migut.L01707 (-I7)
Migut.J00652 (+I7)
MigutL.01707 (-I7)
Migut.J00652 (+I7)
IM62
IM767
