# Supplementary material for: Parallel loss of introns in the ABCB1 gene in angiosperms
Source: BMC Evol Biol. 2017 Dec 4;17:238. doi: 10.1186/s12862-017-1077-x (PMC5716013; doi:10.1186/s12862-017-1077-x)

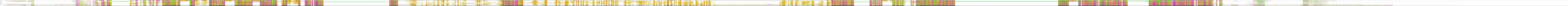

Supplement: Supplementary file 3 — Multiple sequence alignment of monocot ABCB1 genes. A multiple sequence alignment was generated for genomic and, for a few species, cDNA sequences of monocot ABCB1 genes obtained from available whole genome sequence data or sequenced ABCB1 genes. Differentially colored regions indicate exons and introns. (PDF 962 kb) [file 12862_2017_1077_MOESM3_ESM.pdf]
