# Supplementary material for: Parallel loss of introns in the ABCB1 gene in angiosperms
Source: BMC Evol Biol. 2017 Dec 4;17:238. doi: 10.1186/s12862-017-1077-x (PMC5716013; doi:10.1186/s12862-017-1077-x)

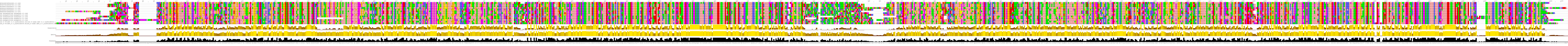

Supplement: Supplementary file 8 — Multiple sequence alignment of selected ABCB proteins from Arabidopsis and rice, and of ABCB1 and ABCB19 from Amborella. A multiple sequence alignment was generated of protein sequences of selected ABCB family members from Arabidopsis and rice, and of ABCB1 and ABCB19 from Amborella. The positions of intron 8-ABCB1 in the variable linker region (~amino acids 681–762) in Arabidopsis and Amborella ABCB1, Amborella ABCB19, and rice ABCB homologs LOC_Os08g05690 and LOC_Os08g05710 are indicated by a black box around the relevant amino acid. (PDF 119 kb) [file 12862_2017_1077_MOESM8_ESM.pdf]
