# Supplementary material for: Parallel loss of introns in the ABCB1 gene in angiosperms
Source: BMC Evol Biol. 2017 Dec 4;17:238. doi: 10.1186/s12862-017-1077-x (PMC5716013; doi:10.1186/s12862-017-1077-x)

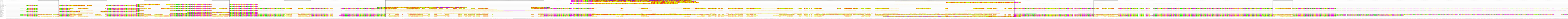

Supplement: Supplementary file 9 — Multiple sequence alignment of genomic sequences from regions of ABCB1 obtained by long-range PCR across all nine introns or amplification across one or a small number of introns. (PDF 337 kb) [file 12862_2017_1077_MOESM9_ESM.pdf]
